# Nanofibrous insulin/vildagliptin core-shell PLGA scaffold promotes diabetic wound healing

**DOI:** 10.3389/fbioe.2023.1075720

**Published:** 2023-04-24

**Authors:** Chen-Hung Lee, Dong-Yi Chen, Ming-Jer Hsieh, Kuo-Chun Hung, Shu-Chun Huang, Chia-Jung Cho, Shih-Jung Liu

**Affiliations:** ^1^ Division of Cardiology, Department of Internal Medicine, Chang Gung Memorial Hospital-Linkou, Chang Gung University College of Medicine, Taoyuan, Taiwan; ^2^ Department of Physical Medicine and Rehabilitation, New Taipei Municipal Tucheng Hospital, Chang Gung Memorial Hospital, New Taipei City, Taiwan; ^3^ Department of Physical Medicine & Rehabilitation, Chang Gung Memorial Hospital, Linkou, Taiwan; ^4^ College of Medicine, Chang Gung University, Taoyuan, Taiwan; ^5^ Institute of Biotechnology and Chemical Engineering, I-Shou University, Kaohsiung, Taiwan; ^6^ Department of Orthopedic Surgery, Bone and Joint Research Center, Chang Gung Memorial Hospital-Linkou, Taoyuan, Taiwan; ^7^ Department of Mechanical Engineering, Chang Gung University, Taoyuan, Taiwan

**Keywords:** core-shell nanofiber, diabetes, wound, insulin, vildagliptin

## Abstract

**Introduction:** Slow wound repair in diabetes is a serious adverse event that often results in loss of a limb or disability. An advanced and encouraging vehicle is wanted to enhance clinically applicable diabetic wound care. Nanofibrous insulin/vildagliptin core-shell biodegradable poly (lactic-co-glycolic acid) (PLGA) scaffolds to prolong the effective drug delivery of vildagliptin and insulin for the repair of diabetic wounds were prepared.

**Methods:** To fabricate core-shell nanofibrous membranes, vildagliptin mixture with PLGA, and insulin solution were pumped via separate pumps into two differently sized capillary tubes that were coaxially electrospun.

**Results and Discussion:** Nanofibrous core-shell scaffolds slowly released effective vildagliptin and insulin over 2 weeks *in vitro* migration assay and *in vivo* wound-healing models. Water contact angle (68.3 ± 8.5° vs. 121.4 ± 2.0°, *p* = 0.006) and peaked water absorbent capacity (376% ± 9% vs. 283% ± 24%, *p* = 0.003) of the insulin/vildagliptin core-shell nanofibrous membranes remarkably exceeded those of a control group. The insulin/vildagliptin-loaded core-shell nanofibers improved endothelial progenitor cells migration *in vitro* (762 ± 77 cells/mm^2^ vs. 424.4 ± 23 cells/mm^2^, *p* < 0.001), reduced the α-smooth muscle actin content *in vivo* (0.72 ± 0.23 vs. 2.07 ± 0.37, *p* < 0.001), and increased diabetic would recovery (1.9 ± 0.3 mm^2^ vs. 8.0 ± 1.4 mm^2^, *p* = 0.002). Core-shell insulin/vildagliptin-loaded nanofibers extend the drug delivery of insulin and vildagliptin and accelerate the repair of wounds associated with diabetes.

## Introduction

Diabetes mellitus is regarded as an important health issue that affects millions of people, predisposing them toward macro-and micro-vascular complications (Misra et al., 2019). Slow wound repair in diabetes is a serious adverse event that often results in loss of a limb or disability (Menke et al., 2015). Diabetic wounds leading to lower extremity amputation are one of the most common adverse outcomes associated with diabetes mellitus, and impairment of their healing leads to high mortality (Armstrong et al., 2020). Hyperglycemia in patients with diabetes has been found to disrupt the balance of pressure-induced vasodilation due to the dysfunction of endothelial cells, to interfere with the processes of re-epithelialization as a result of protein synthesis, cell migration and proliferation (Lima et al., 2017), and to increase free radical damage that is caused by reducing antioxidant activity (Sedighi et al., 2014).

Insulin, a peptide hormone and growth factor, which is critical in patients with diabetes because it promotes both the healing of injured skin by stimulating the signal of proliferation and migration and growth factor release through the stimulation of endothelial cells, fibroblasts, and keratinocytes (Gurtner et al., 2008; Brem and Tomic-Canic, 2007; Wertheimer et al., 2001). Inhibitors of dipeptidyl peptidase (DPP)-4 have been developed based on the gut-derived glucagon-like peptide-1, one of specific antidiabetic hormones because it promotes insulin release and suppresses glucagon secretion (Lambeir et al., 2003; Yin et al., 2022). However, presently prescribed anti-diabetic medications are associated with poor adherence and poor compliance by patients due to their many concerns, including hypoglycemia, edema, weight gain, gastrointestinal derangements and nausea (Osadebe et al., 2015). Thus, advanced methods for controlling sustained drug release and minimizing associated side-effects are greatly desired.

An ideal means of administering drugs to treat diabetic wounds would allow for preferential targeting of wound locations, extended therapeutic applications, and prolonged activity of the drug *in vivo*. Extensive research is being conducted on electrospun nanofibers that closely resemble the native extracellular matrix (ECM) structure, with the aim of using them in tissue engineering and biomedical applications (Lee et al., 2003). Biodegradable core-shell nanostructures that are prepared by the coaxial electrospinning method have been fabricated as medical implants, into which bioactive molecules are integrated (Jiang et al., 2005; Huang et al., 2006). The coaxial electrospinning technique provides the advantage of encapsulating biomolecules at the core to avoid the contact with organic solvents and prevent loss of their bioactivity (Lu et al., 2016; Zare et al., 2021). While most studies of nanofibers delivered only single drug, the core-shell structured nanofibers developed in this research delivered two biomolecules at the same time. Furthermore, to the best of authors’ knowledge, no previous works have investigated the co-delivery of water-soluble vildagliptin (Waghulde and Naik, 2017; Waghulde et al., 2019) and bioactive insulin simultaneously.

The topical wound dressing with a core-shell biodegradable PLGA nanofibers that is encapsulated with vildagliptin and insulin is hypothesized to promote diabetic wound healing. Nanofibrous insulin-loaded core-shell biodegradable scaffolds with or without vildagliptin were developed by the coaxial method of electrospinning. The structure and morphology of the electrospun products was evaluated using scanning electron microscopy (SEM) and transmission electron microscopy (TEM) following the electrospinning procedure. The effects of nanofibrous core-shell insulin-loaded membranes with or without vildagliptin on the recovery of diabetic wounds was examined using immunofluorescence and histology.

## Materials and methods

### Core-shell nanofibrous scaffolds

The material poly (lactic-co-glycolic acid) (PLGA) (Resomer RG 503, Boehringer, Germany) with a lactide:glycolide ratio of 50:50 and a mean molecular weight of 33,000 Da was utilized herein. Insulin glargine, a sterile solution of insulin (Lantus), was obtained from Sanofi-Aventis Inc (Frankfurt, Germany). Vildagliptin (C_17_H_25_N_3_O_2_) and 1,1,1,3,3,3-hexafluoro-2-propanol (HFIP) were obtained from Sigma-Aldrich (Saint Louis, MO, United States).

Two nanofibrous core-shell scaffolds were fabricated using a special well-designed coaxial electrospinning device that concurrently delivered two solutions from two separate feeding tubes to an aluminum plate (Hsu et al., 2016). Based on our preliminary results, a 1:3 ratio of vildagliptin and PLGA shell design has sufficient core-shell scaffold performance. A mixture of vildagliptin (210 mg) and PLGA (630 mg) from group A (Vildagliptin 25%) or PLGA (840 mg) from group B (Vildagliptin 0%) was dissolved in 3,000 µL HFIP to prepare shell solution. Use 1,000 µL of insulin glargine (equivalent to 3.64 mg) (100%) as the core solution for the feeding syringes. During electrospinning, two independently controlled pumps delivered both liquids onto the aluminum plate at volumetric flow rates of 5 μL/min for the core insulin solution and 15 μL/min for the shell vildagliptin/PLGA solution. The electrospinning studies were conducted at 25°C. Electrospun nanofibers were put in a laminar flow hood for 72 h and then were maintained at 4°C.

### Porosity

The density of the two core-shell electrospun nanofibers were measured using mass divided by volume. The porosity of both core-shell nanofibrous membranes was assessed using the calculation as followed (Lee et al., 2022a).

$$Pore\;\left(\%\right)=1-\sigma_{drug\left(s\right)/PLGA}\;/\sigma_{PLGA}.\;\sigma =density.$$ (1)

### SEM and TEM

The SEM images of 100 randomly selected areas of fiber (Lee et al., 2015) were used to assess the size distribution and pore area of the electrospun nanofibers. The analysis was conducted using ImageJ software from the National Institutes of Health in the United States. Additionally, the morphology of the core-shell nanofibers was examined using TEM (JEOL JEM-2000EXII, Japan) in three separate instances.

### Mechanical properties of core-shell nanofibers

Mechanical properties were evaluated via a Lloyd tensiometer (AMETEK, United States) - tensile strength (the ratio of breaking force (N) to the sample area of cross section (mm^2^), and is measured in MPa) and elongation at break (the percentage increase in length at the break point (mm) divided by the initial length (mm) and multiplied by 100) of the nanofibrous test samples (n = 3).

### Wetting angles (contact angle of water)

To measure the wetting angle of the nanofibers, a water contact angle analyzer featuring a video monitor (First Ten Angstroms, United States) was used. Three samples, each with dimensions of 10 mm × 10 mm, were taken from both sets of membranes and placed on the test plate. Pure water was then carefully released onto the surface of each sample.

### Water absorption capacity

The two nanofibers at 0.5, 1, 2, 3, 8, and 24 h were gotten by the water content formula. The water absorption capacity (%) was analyzed as follows.

$$Water\;absorption\;capacity\;\left(\%\right)=\left(W\;–W_{0}\right)/W_{0}\times 100$$ (2)

where W_0_ and W is the sample weight before and after placing in PBS for 3 min after removing surface water with absorbent paper (n = 3).

### *In vitro c*oncentrations of vildagliptin and insulin released

A release pattern of vildagliptin or insulin from the membranes were calculated using an elution method. Membranes (10 mm × 10 mm) were analyzed (n = 5) in 1,000 µL of phosphate-buffered saline (PBS) and were stored at 37°C before the eluent was collected for analysis. One mL PBS was replaced once a day for 30 days. Hitachi L-2200 Multisolvent Delivery System, an HPLC assay (Japan), with an XBridge C_18_ 5 μm, 4.6 × 150 mm HPLC column (Waters) for vildagliptin separation, was used to evaluate the profile of vildagliptin release from the fabricated nanofibers. The bioactivity of insulin was evaluated via an human insulin solid-phase sandwich ELISA (enzyme-linked immunosorbent assay) (Thermo Fisher Scientific, United States).

### Migration assay of endothelial progenitor cells (EPCs) using eluate from both groups

Transwell filters (Costar, United States) with pores (8.0 µm in diameter) were applied for EPC migration assay, as reported elsewhere (Lee et al., 2021; Lee et al., 2022b). The cells were acquired from Molecular Pharmacology Laboratory (Chang-Gung University, Taiwan). Data were gotten from five randomly selected regions using the eluent released from insulin/vildagliptin-eluting PLGA or insulin/PLGA scaffolds at different time point (n = 4).

### Diabetic wound closure assessment

All operations related to diabetic model were carried out following the confirmation of Institutional Animal Experiment Committee of Chang Gung University (CGU 14–045). Induction of diabetes was performed using fourteen Sprague-Dawley rats with streptozotocin (STZ) (Sigma, United States). Seven days following intraperitoneal injections of 70 mg/kg of STZ, diabetic status was determined by identifying hyperglycemia (≧300 mg/dL) before diabetic wound assessment was performed.

Seven animals were prepared with insulin/vildagliptin-eluting PLGA nanofibrous scaffolds as group A, and the other seven were prepared with insulin/PLGA scaffolds as group B.

Following being anesthetized, a sterile 8.0 mm plastic template was put on mid-back of the diabetic rats, and an excisional wound with full-thickness was created by taking away the skin based on the template. Wound edges were examined using glass microscope slides and assessed for size by planimetry using ImageJ. The entire wound was excised down to the fascia on day 14 along with a margin (5 mm) of uninjured skin. Prior to performing the frozen section with cryostats, the sample was placed in a microtome using an optimal cutting temperature compound.

### Immunofluorescence

Samples were washed extensively in PBS buffer containing 0.05% (v/v) Tween-20 (PBST) and 2% bovine serum albumin blocking at room temperature for 30 min for immunofluorescence staining. The samples were then got along with primary antibodies against α-smooth muscle actin (α-SMA) (1/500, ab5694, Abcam) at least 8 h at 4°C. After being treated with AF 546 goat anti-rabbit secondary antibodies (1/500, Life Technologies), the samples were left to stain for a minimum of 8 h in a cool room at 4°C. On the following day, 4,6-Diamidino-2-phenylindole (DAPI) (nuclear stain, 1/2000, Sigma) was added and the samples were then made ready for visualization using a Leica confocal microscope (n = 4).

### Statistics and data analysis

Data were analyzed in SPSS software (version 17.0 for Windows; SPSS Inc., United States). Data are represented as mean ± standard deviation (SD). For continuous variables with a normal distribution, means were compared using an unpaired Student’s t-test. Otherwise, a Mann-Whitney *U* test was performed. Statistical significance was determined by a *p*-value less than 0.05.

## Results and discussion

### Morphology of electrospun coaxial nanofibers

**FIGURE 1:**
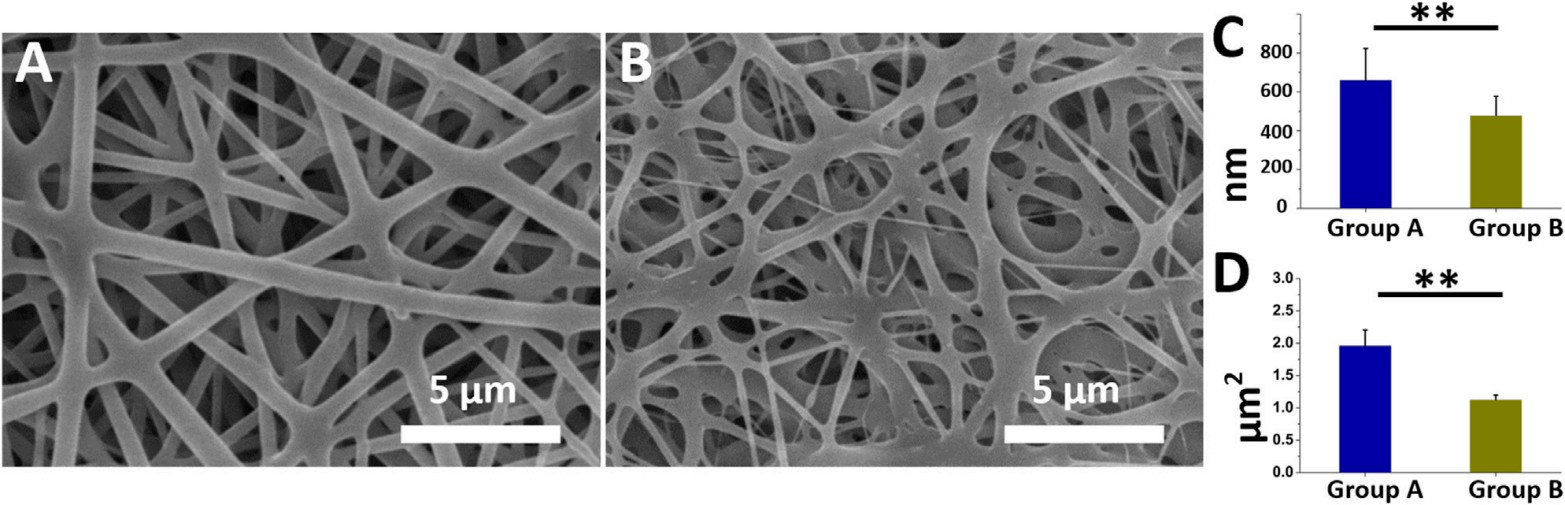
Two electrospun nanofibers were examined utilizing SEM. Appearance of insulin/vildagliptin-eluting **(A)**, and insulin/PLGA nanofibers **(B)**. Diameters **(C, D)**, and E and pore areas (F, G, and H) in two groups were determined using ImageJ (Scale bar: 5 μm). ** *p* < 0.01.

Figures 1A, B present the morphologies and diameter distributions of the coaxially electrospun nanofibers (magnification ×3,000) that had been loaded with insulin/vildagliptin-eluting PLGA (Group A) or insulin/PLGA nanofibers (Group B) (Supplementary Figure S1). The pictures can be clearly noticed that the vildagliptin-loaded core-shell nanofiber membranes clearly had a larger diameter (662 ± 162 nm) than the insulin/PLGA nanofibers (478 ± 99 nm) (*p* = 0.007) (Figure 1C). The pore area of the coaxial nanofibrous insulin/vildagliptin-eluting PLGA membranes [1965 ± 238 × 10^3^ nm (Menke et al., 2015)] significantly exceeded that of the insulin/PLGA membranes [1,128 ± 71 × 10^3^ nm (Menke et al., 2015)] (*p* < 0.001) (Figure 1D). The value of the core-shell group A membranes porosity (88.9 %± 2.0%) was higher than that of the group B scaffolds (78.7 %± 2.0%) (*p* = 0.004).

The mixture of vildagliptin with PLGA might have increased the stability of bending and stretching properties and the jet during electrospinning, increasing the diameter of the fabricated fibers over that of the crude PLGA fibers, and yielding the smooth morphology and structure of the consequently formed nanofibrous scaffolds. Nanofibrous scaffolds that were prepared by the electrospinning method mimicked the biological functions or structure of the natural extracellular matrix (ECM), offering mechanical strength to the structure of cells and guiding cellular migration (Ingavle and Leach, 2014). Additionally, the nanofibrous structures had an elevated ratio of surface area-to-volume and high porosity, favoring cellular proliferation, migration, and adhesion (Khorshidi et al., 2016).

### Fluorescent images, TEM images and mechanical properties

**FIGURE 2:**
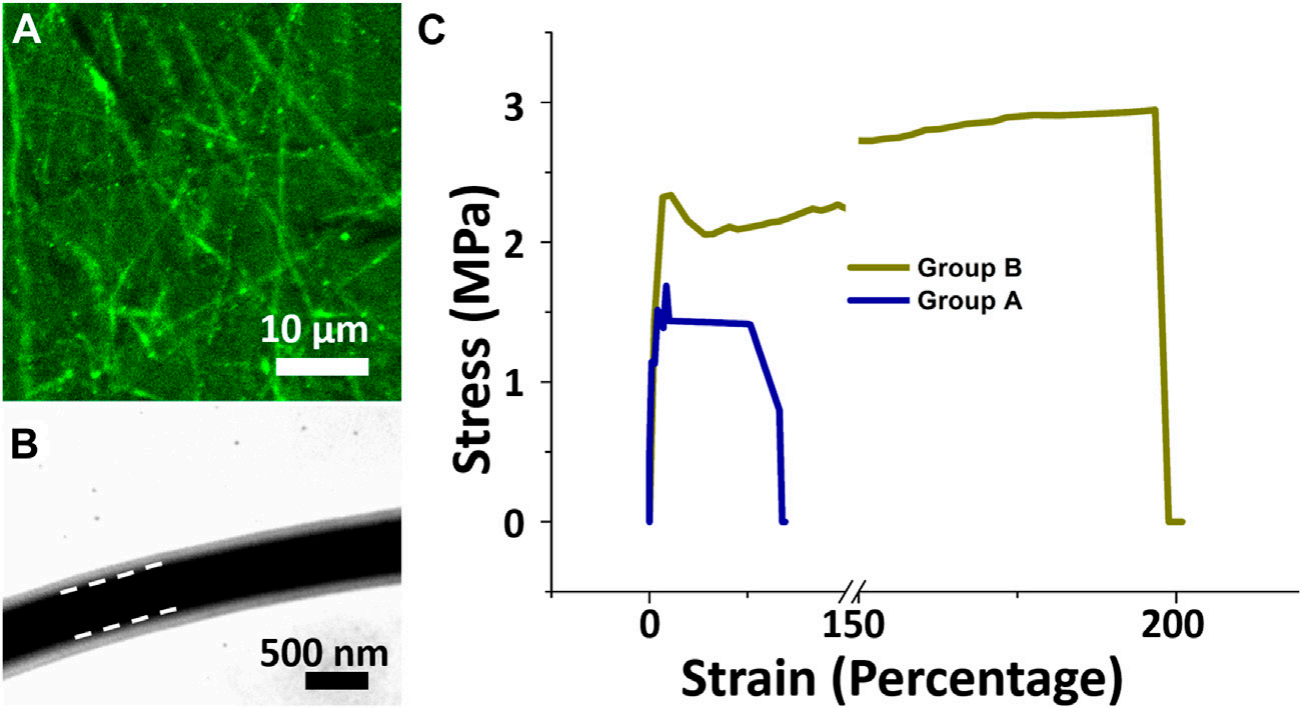
Laser scanning confocal microscopic and transmission electron microscopic images. Core-shell nanofibers with reGFP as core and PLGA as shell were fabricated **(A)**. Core-shell nanofibrous scaffolds with liquid solution between white dashed lines **(B)**. Stress-strain curves of core-shell of both groups **(C)**. Stress (MPa) and percentage strain in group A were less than in group B..

To verify the core-shell structure, nanofibers containing recombinant green fluorescence protein (reGFP) at the core and pure PLGA as the shell were fabricated through co-electrospinning. The reGFP image in Figure 2A observed using a fiuorescence microscope shows a continuous and clear fluorescent signal (green string-like filaments), indicating the presence of a highly bioactive protein at the core. The TEM image in Figure 2B indicates that the fabricated nanofibers had a distinct core-shell structure and a density that differed between the two-white dashed. These images confirm the core–shell structure of the spun nanofibers and the encapsulation of insulin within the polymer PLGA shell.

As a dressing for wound repair, a scaffold should be able to support cell growth and resist stress and movement. Figure 2C displays the mechanical properties of both electrospun core-shell nanofibrous scaffolds. The mean tensile strengths for nanofibrous membranes with insulin/vildagliptin-eluting (Group A) and insulin/PLGA (Group B) were about 1.23 ± 0.19 MPa and 2.90 ± 0.05 MPa, respectively (*p* < 0.001). The nanofibrous scaffolds in Group A had less elongation at breakage (29.9 %± 3.0% vs. 180.4 % ± 15.1%, *p* < 0.001) than those in group B. In a drug-loaded polymeric composite, the polymer is the component that resists the external force and supports plastic extension. The mixture with vildagliptin decreased the content of PLGA in the composite herein. The maximum elongation and strength at breakage of PLGA nanofibers were reduced accordingly (Wang et al., 2021a; Wang et al., 2021b).

### Surface hydrophilic properties

**FIGURE 3:**
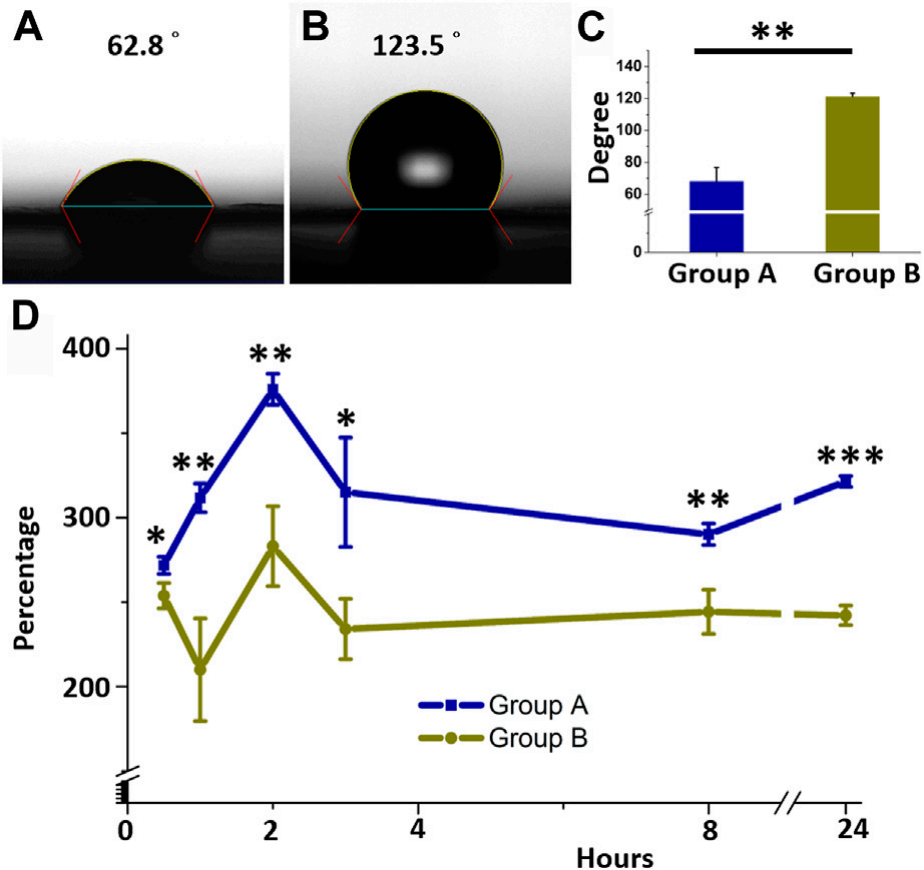
Water contact angles and water uptake. Core-shell insulin/PLGA with vildagliptin **(A)** or without vildagliptin nanofibrous scaffolds **(B)**. Measured contact angles of two groups **(C)**. Variance between water absorbent capacities of two nanofibrous groups over time **(D)**. *p < 0.05, **p < 0.01, ***p < 0.001.

Figure 3 represents the contact angles of water on core-shell insulin/Vildagliptin PLGA nanofibers with vildagliptin (Figure 3A) or insulin/PLGA nanofibers with no vildagliptin (Figure 3B). The results of the core-shell insulin/vildagliptin-loaded and the insulin/PLGA nanofibrous scaffolds were 68.3 ± 8.5° and 121.4 ± 2.0°, respectively. Mixing with vildagliptin significantly increased the hydrophilicity of the core-shell insulin/PLGA nanofibrous membranes (Figure 3C) (*p* = 0.006). Figure 3D shows the percentages of water absorption capacity in the both core-shell nanofibers following PBS immersion for 0.5, 1, 2, 3, 8, and 24 h. Group A nanofibrous membranes had the highest water content (379 %± 9%) at 2 h and maintained a higher water content (>272%) during the first 24 h. Without vildagliptin, the capacity of group B nanofibers for storing water was always lower than that of group A and group B nanofibrous scaffolds also had their highest water content at 2 hours (283 %± 24%) (Supplementary Table S1, water absorption capacity of both groups) Due to the capillary effect of the numerous pores, the water uptake of nanofibers increased with time after immersion in an aqueous environment. The uptake reached peak values at 2 h, and gradually diminished and leveled off thereafter. The modification of the wetting properties of a polymer surface can be achieved by the integration of hydrophilic moieties. This is attributed to the increased polarity of the polymer and the subsequent reduction in the angle of contact with water (Kiss et al., 2002; Yilgor et al., 2012). Consequently, the inclusion of the water-soluble hydrophilic drug, vildagliptin, facilitated the water absorption of nanofibers (Hess et al., 2011; Lee et al., 2022c). Nanofibrous scaffolds in Group A thus exhibited a greater water uptake capability than those in Group B. As a scaffold for skin dressings, surface conditions significantly affect the response of cells to the water content of biomaterials. The core-shell nanofibrous scaffolds demonstrated improved wettability, which in turn would promote cellular activities such as proliferation, differentiation, and attachment, ultimately leading to tissue regeneration (Wu et al., 2015; Wang et al., 2016).

### *In vitro* release curves of vildagliptin and insulin and EPCs migration assay

**FIGURE 4:**
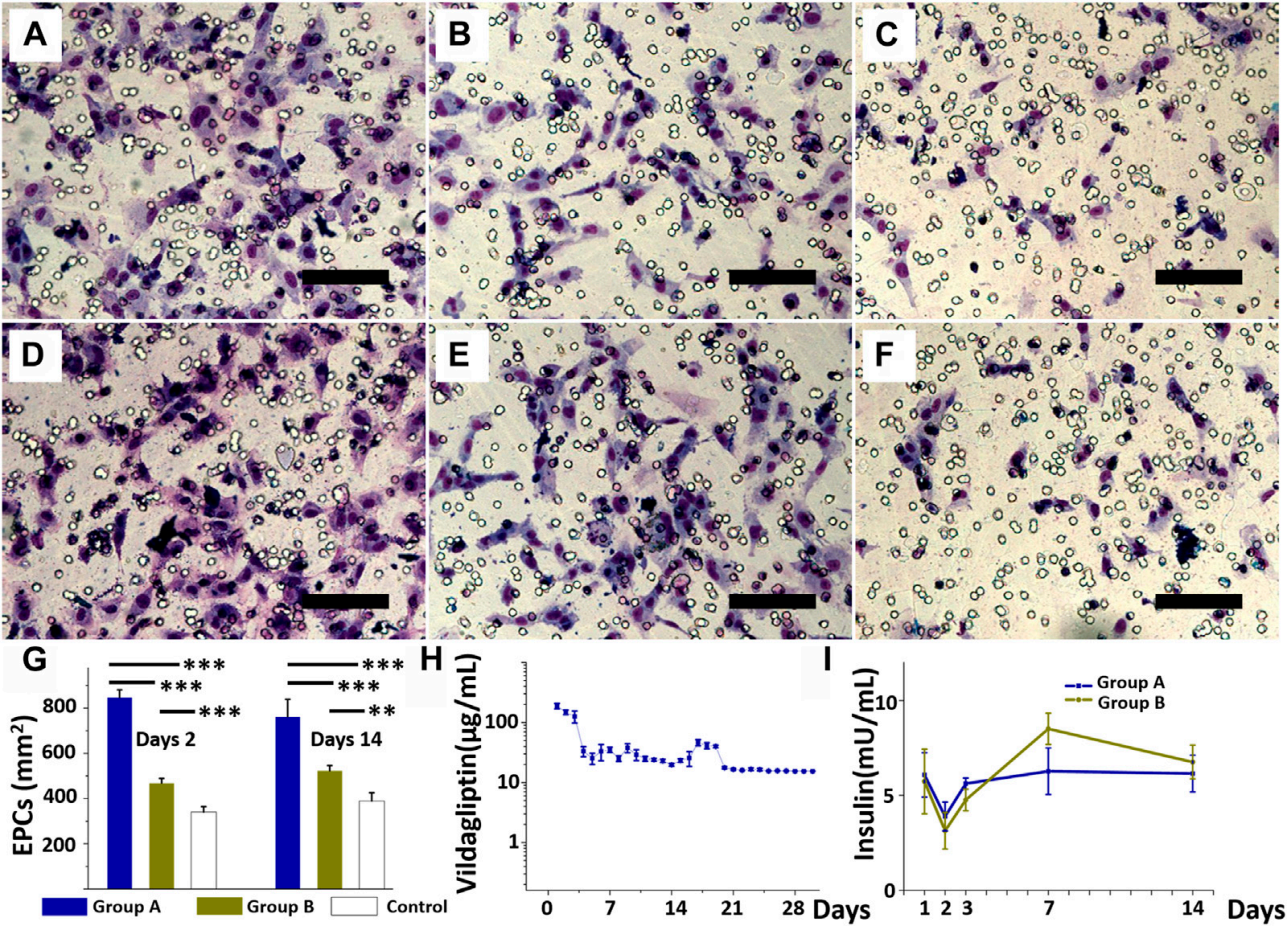
EPCs treated with the eluate on day 2 **(A, B, and C)** and eluate on day 14 **(D, E, and F)** from insulin/vildagliptin-eluting group **(A and D)**, EPCs treated with eluents from insulin/PLGA group **(B and E)**, and control group **(C and F)**. EPC migration in three groups treated with days 2 or days 14 eluents **(G)**. *In vitro* release of vildagliptin in group A for 30 days **(H)**, and insulin in groups A and B for 14 days, with no significant difference **(I)**. (Scale bar = 100 µm). ** *p* < 0.01, *** *p* < 0.001.

Data from the EPC transwell migration assay (Figures 4A–F), more cells migrated after treatment with the eluate on day 2 (Figures 4A–C) and day 14 (Figures 4D–F) in group A (day 2 eluate: 847 ± 33 cells/mm^2^; day 14 eluate: 762 ± 77 cells/mm^2^) than after treatment in group B (day 2 eluate: 469 ± 20 cells/mm^2^; day 14 eluate: 424 ± 23 cells/mm^2^) or DPBS (day 2 eluate: 340 ± 24 cells/mm^2^; day 14 eluate: 389 ± 36 cells/mm^2^) (all *p* < 0.001) (group B vs. control, *p* < 0.001 for day 2 eluate, and *p* = 0.005 for day 14 eluate). Figure 4G shows that cell migration with vildagliptin and insulin was superior to insulin alone or DPBS alone (all *p* < 0.001). Figure 4H plots release curves *in vitro* of vildagliptin every day. The core-shell insulin/vildagliptin-eluting PLGA nanofibers delivered vildagliptin constantly for 30 days, with an rapid release of the beginning on day one (188 ± 20 μg/mL), and then continuously release (at least 16 μg/mL) until day 30. Figure 4I plots the daily release profile of insulin *in vitro*. Both core-shell nanofibers delivered stable and sustained insulin concentrations from day 1 (6.1 ± 1.2 vs. 5.7 ± 1.7mU/mL, *p* = 0.789) to day 14 (6.2 ± 1.0 vs. 6.8 ± 0.9mU/mL, *p* = 0.469). (See Supplementary Table S2). The augmented hydrophilicity of vildagliptin-loaded nanofibers may enhance insulin release concentration from day 1 to day 3 when compared with the non-vildagliptin-loaded group.

EPCs contribute to angiogenesis and vascularization during wound recovery stage but impaired EPCs recruitment in cases of diabetes (Loomans et al., 2004; Ambasta et al., 2017). Under hyperglycemic conditions, endothelial progenitor cells (EPCs) are functionally impaired in their ability to migrate, mobilize, and integrate/differentiate into existing vasculature (Saboo et al., 2016; Hu et al., 2018). DPP4 inhibitors, such as vildagliptin and sitagliptin, induce pro-angiogenic and anti-apoptotic effect in EPCs by improving glucose tolerance and restoring normal biologic functions (Fiordaliso et al., 2016; Liu et al., 2016; Lee et al., 2022c). Insulin treatment has also been shown to enhance EPC proliferation and their angiogenic activity (Zhao et al., 2011). Therefore, delivery of insulin and vildagliptin using core-shell nanofibrous membranes to promote diabetic wound healing may be due to increased EPC activity rather than increased circulating EPCs and their homing to the wound area. The internal structure and composition of nanofibers critically determine whether certain drug-release profiles can be achieved. The high porosity of the fibrous scaffolds and its large contact area for dissolution make nanofibers promising candidates for drug release (Braghirolli et al., 2014; Potrč et al., 2015). To prolong drug release, either core-shell nanofibers, sandwich-type nanofibrous meshes, or multi-drug-loaded layers can be used (Lee et al., 2020). Supplementary Figure S2 displays the results of FTIR measurements of the vildagliptin-loaded PLGA and pure PLGA core-shell nanofibers. The FTIR data of vildagliptin/PLGA nanofibers elucidated important wide spikes between 3,110 and 3,700 cm^−1^, demonstrating N–H stretching and OH vibrations. The other vibrational peak at 2,250 cm^−1^ was indicated to nitrile stretching vibrations (Harsha et al., 2015). An overlap of typical peaks was noticed, illustrating reasonable stability and incorporation of the vildagliptin during core-shell electrospinning of nanofibers using HFP solvent.

In summary, the biocompatible core-shell nanofibers that are prepared in this work had effective concentrations of vildagliptin and insulin for 30 and 14 days, respectively.

### Wound healing and histological examination

**FIGURE 5:**
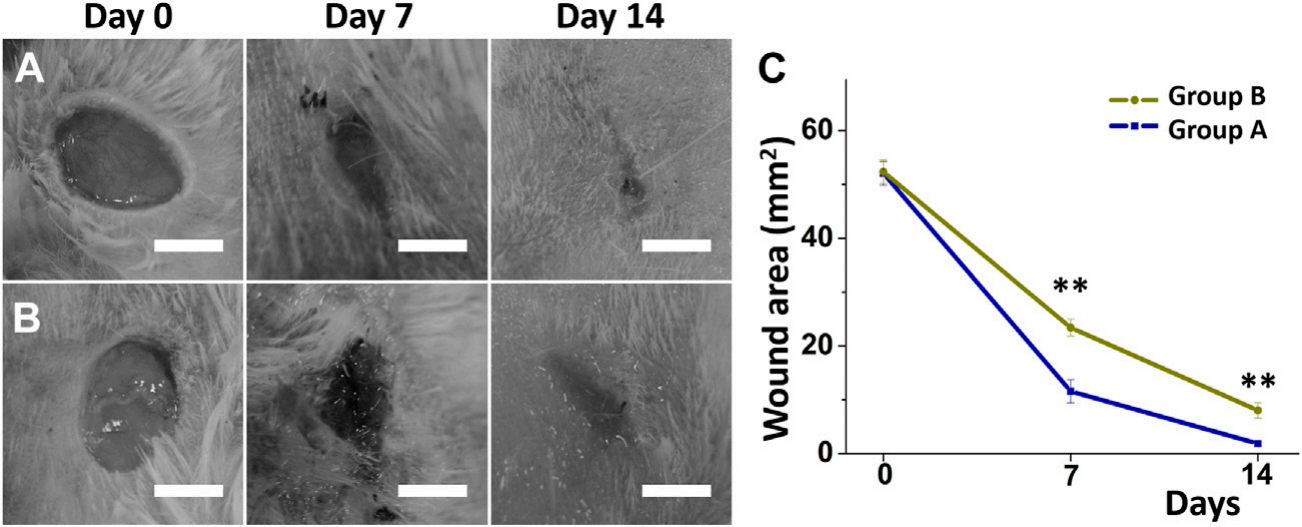
Diabetic wound recovery following management with insulin/vildagliptin-eluting scaffolds (group A) **(A)** or insulin/PLGA (group B); **(B)** nanofibrous membranes on day 0, day 7, and day 14. The wound area was assessed on different days **(C)**. (Scale bar = 5 mm). ** *p* < 0.01.

Figure 5 presents representative images of a healing wound on a diabetic animal from both treatment groups using different nanofibers on days zero, seven, and 14 after surgery with core-shell nanofibrous membranes. The wound regions in the two treatment groups were similar on the days zero (52.1 ± 2.2 vs. 52.4 ± 2.2 mm (Menke et al., 2015), *p* = 0.881). However, wounds in group A (insulin/vildagliptin-eluting PLGA core-shell membranes) were significantly smaller than those in group B (insulin/PLGA) on days seven and 14 (Day 7: 11.6 ± 2.2 vs. 24.4 ± 1.6 mm (Menke et al., 2015), *p* = 0.002; Day 14: 1.9 ± 0.3 vs. 8.0 ± 1.4 mm (Menke et al., 2015), *p* = 0.002) (Figure 5C). On the 0th, seventh, and 14th days, the average blood glucose values of group A were consistent with those of group B (all *p* > 0.05) (Supplementary Table S3).

Skin is a complex and dynamic organ, whose integrity following skin injury is restored in a process that depends on the interaction of fibroblasts, neovascularization, and biomacromolecules (Arthur and Luciano, 2000; Barrientos et al., 2008). The exogenous application of insulin has been demonstrated to stimulate cellular migration and diabetic wound recovery (Abdelkader et al., 2016; Lee et al., 2020). DPP-4 inhibitors favor diabetic wound healing by promoting the recruitment of EPCs and supporting the final scaffolding of wounds (Saboo et al., 2016). DPP-4 inhibitors also support angiogenesis and have a wide range of effects, including optimizing the immune response and decreasing the adverse effect of hypoxia in prolonged diabetes wounds (Huang et al., 2012). Therefore, dressing a diabetic rat wound with core-shell insulin/vildagliptin-eluting PLGA nanofibrous scaffolds healed faster than the core-shell insulin/PLGA group after 2 weeks.

**FIGURE 6:**
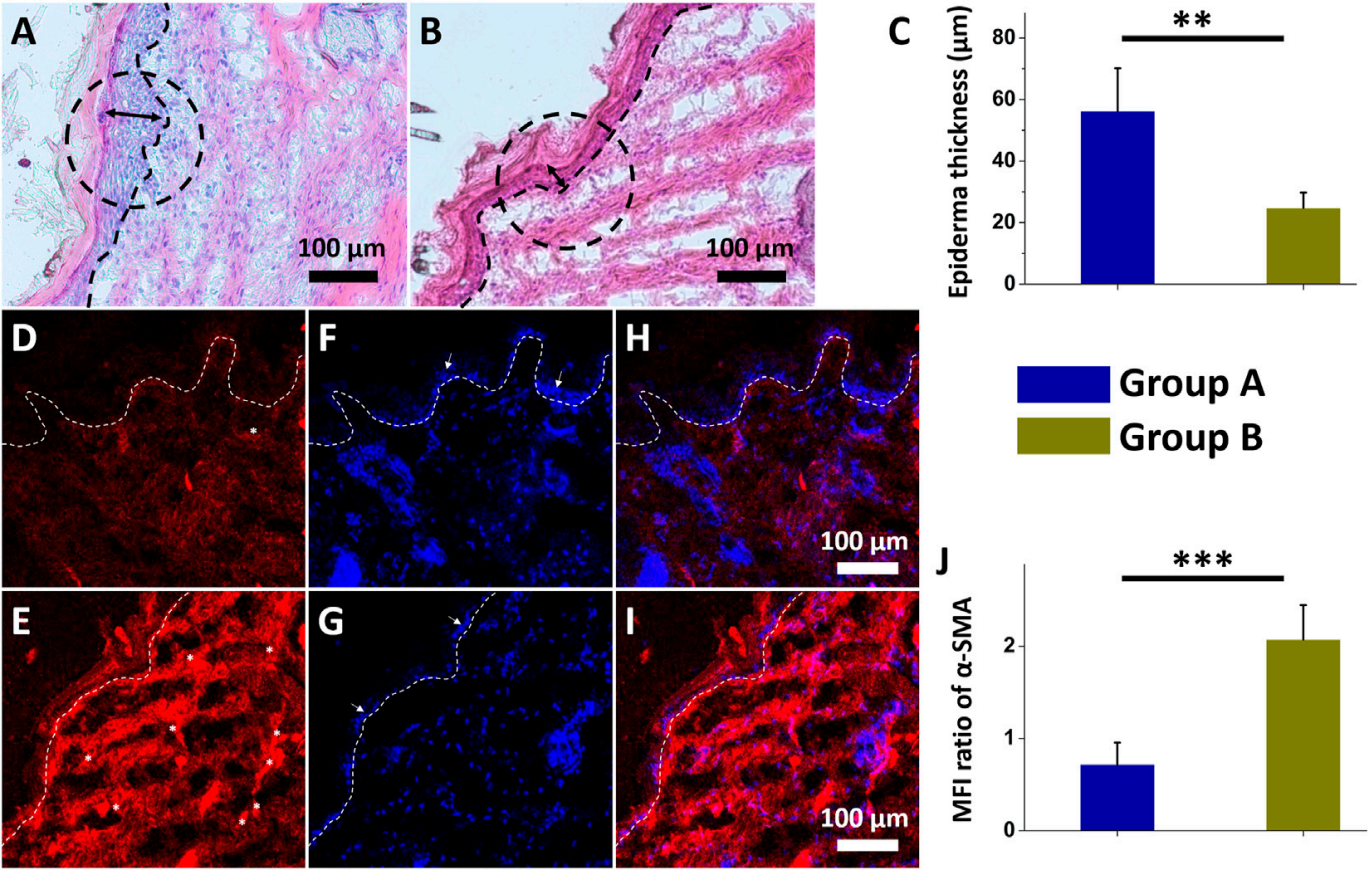
Histological images of insulin/vildagliptin-eluting PLGA group (Group A) **(A)** and insulin/PLGA (Group B) **(B)** after two weeks. The treated wound sections showed deeper epidermis (double arrow area and black circular dashed line) and infiltration of the cells on dermis area (below black dot line) in group A **(C)**. Immunofluorescence of α-SMA in group A **(D)** and group B **(E)**. Photos of DAPI-labeled nuclei (blue) **(F and G)**, and merged images **(H and I)** of two groups. The white arrow **(F and G)** indicated the epidermis, consistent with the above results **(C)**. α-SMA (white asterisk) immunocytochemistry was analyzed relative to that of DAPI using MFI **(J)** (Scale bar = 100 μm). ** *p* < 0.01, *** *p* < 0.001 (n=4).

The histological photos reveal that nanofibrous core-shell insulin/vildagliptin-eluting PLGA membranes (Figure 6A) increased cell proliferation in the dermis area than the insulin/PLGA nanofibers group (Figure 6B). Treatment with group A scaffold for 2 weeks showed a thicker epidermis in the cross-sectional wound than achieved in group B (56.3 ± 13.9 µm vs. 24.8 ± 4.9 µm, *p* = 0.005) (Figure 6C). The results of immunostaining for α-SMA expression in both groups (Figure 6D for group A; Figure 6E for group B) revealed a significant difference when normalized to those of DAPI nuclear staining (group A: 0.72 ± 0.23 vs. group A: 2.07 ± 0.37, *p* < 0.001).

Diabetic wound healing are typically associated with reduced granulation tissue thickness, delayed re-epithelialization, decreased infiltrated cells, reduced matrix density, and impaired angiogenesis (Broadley et al., 1988; El Gazaerly et al., 2013). High DPP4 levels in diabetic wounds impair wound healing by sustaining an inflammatory status (Baticic Pucar et al., 2017). The vildagliptin, one of DPP4 inhibitor, that was slowly and sustainably released from core-shell nanofibrous scaffolds improves wound healing herein may be due to the possible effect of increasing the migration rate of keratinocytes and fibroblasts to diabetic wound regions, promoting re-epithelialization of the wound (Saboo et al., 2016; Adeghate et al., 2017; Lee et al., 2022c).

α-SMA is the actin isoform that dominates in smooth-muscle cells and has an vital role in fibrogenesis (Storch et al., 2007). Myofibroblasts are morphologically and metabolically distinctive fibroblasts that express α-SMA, and their activation has a critical role in the expansion of the fibrotic tissue and the release of large amounts of extracellular component proteins (Langevin et al., 2010). The presence of more myofibroblasts (expressing α-SMA protein) later in wound recovery is a specific hallmark of impaired wound recovery, as most of them disappear during granulation cell apoptosis (Desmouliere et al., 1995; Toriseva et al., 2012). The experimental data in this study suggest that the biomolecule-loaded scaffold can greatly reduce the expression ofα-SMA protein by releasing vildagliptin and insulin. The use of nanofibrous scaffolds, which release vildagliptin and insulin, therefore accelerates wound closure and promotes the healing of diabetic wounds.

Despite its strengths and contributions, this study has certain limitations that warrant further investigation. First, many molecular and cellular response differences between acute and chronic wounds have been demonstrated (George Broughton et al., 2006; Enoch and Price, 2004). For example, chronic wounds often exhibit persistent inflammation, delayed re-epithelialization, suboptimal granulation tissue formation, and poor fibroblast infiltration (Martin and Nunan, 2015). Therefore, diabetic wounds are often used as one of the *in vivo* models of chronic wounds developed by subjecting acute wounds to the main clinical causes of chronic wounds (Loots et al., 1998; Blakytny and Jude, 2006). Second, when is the optimal period for wound management after diabetes induction? According to the literature, 2–3 days (Kishore et al., 2017; Siracusa et al., 2018), 7 days (Gallagher et al., 2007; Yuan et al., 2018), or 2–3 weeks (Lee et al., 2012; Ramachandran et al., 2012) following the induction have been used. Finally, after 7-day protocol to induce experimental diabetes, skin wounds may not have all the features of chronic lesions, and the imperative to conduct further research is needed in order to attain a more comprehensively understand of the mechanisms associated with the diabetic wound healing.

## Conclusion

Resorbable and biocompatible core-shell insulin/vildagliptin-eluting nanofibrous membranes that sustainably release insulin and vildagliptin for 2 weeks and at least 4 weeks, respectively, were developed using electrospinning. These vildagliptin-loaded PLGA scaffolds had less hydrophobicity and water absorbent capacity than PLGA membranes. The functional and constant delivery of vildagliptin increased the degree of migration of EPCs and enhanced diabetic wound recovery. The core-shell insulin/vildagliptin-eluting nanofibrous scaffolds increased epidermal thickness, promoted wound closure, and reduced α-SMA expression relative to those in the insulin/PLGA group. The insulin/vildagliptin membrane based on electrospun core-shell PLGA is biomechanically and biologically effective in promoting diabetic wound healing and reducing fibrotic effects, which is beneficial to the restoration of cell function and granulation.

## Data Availability

The original contributions presented in the study are included in the article/Supplementary Material, further inquiries can be directed to the corresponding author.
