# Supplementary material for: Nanofibrous insulin/vildagliptin core-shell PLGA scaffold promotes diabetic wound healing
Source: Front Bioeng Biotechnol. 2023 Apr 24;11:1075720. doi: 10.3389/fbioe.2023.1075720 (PMC10164987; doi:10.3389/fbioe.2023.1075720)
Supplement: Supplementary file 1 [file DataSheet1.pdf]

Supplemental Information

Nanofibrous Insulin/Vildagliptin Core-shell PLGA Scaffold Promotes Diabetic Wound Healing

***To whom correspondence and reprint requests should be addressed:**

Shih-Jung Liu Email: shihjung@mail.cgu.edu.tw
Chen-Hung Lee E-Mail: chl5265@gmail.com
Chia-Jung Cho E-mail: ppaul288@isu.edu.tw

List of Supplemental Information:

1. Figure S1. Data representation of diameter and pore area.
2. Figure S2. The FTIR measurements were conducted on both the pure PLGA and the vildagliptin-loaded PLGA core-shell nanofibers.
3. Table S1. Percentage water content
4. Table S2. Insulin concentration in both groups.
5. Table S3. Blood sugar in both groups

Supplemental 1. Data representation of diameter and pore area.

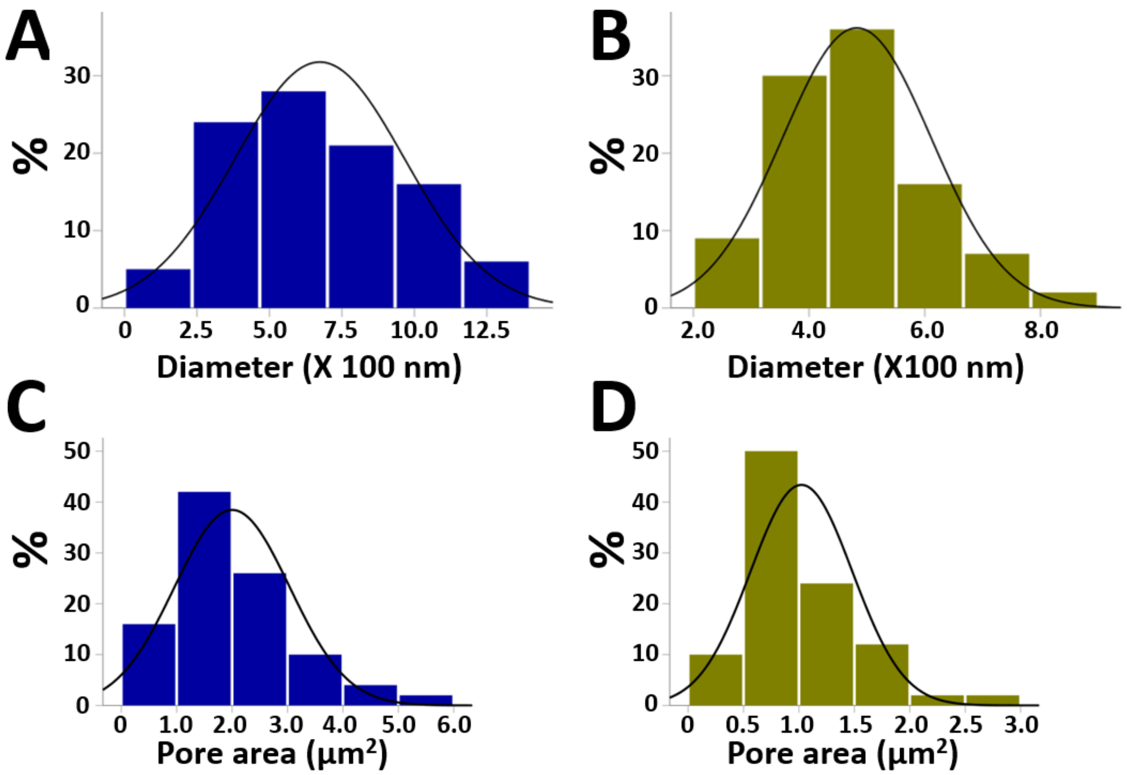

Figure S1. The diameter (A and B) and pore area (C and D) of both electrospun core-shell nanofibers. Morphology of insulin/vildagliptin-eluting (A and C), and insulin/PLGA nanofibers (B and D).

Supplemental 2. The FTIR measurements were conducted on both the pure PLGA and the vildagliptin-loaded PLGA core-shell nanofibers.

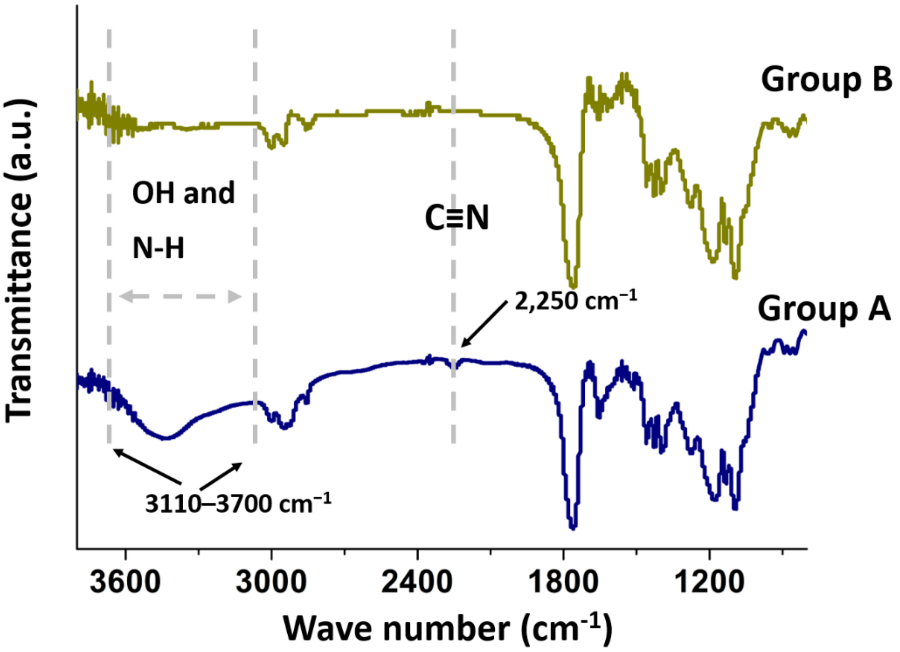

Figure S2. The FTIR analysis of the vildagliptin core-shell PLGA nanofibers revealed significant peaks in the range of 3110–3700 cm^{-1} (broad), indicating stretching vibrations of OH and N–H. Additionally, another peak at 2,250 cm^{-1} was observed, which was attributed to the stretching vibrations of nitrile.

Supplement 3: Percentage water content

Table S1. Percentage water content in both fabricated core-shell nanofibers over 24 hours

Time (hour)	Insulin/vildagliptin-eluting PLGA group, %	Insulin/PLGA group, %	*p* value
0.5	272 ± 5	254 ± 8	0.027
1	312 ± 9	210 ± 30	0.005
2	376 ± 9	283 ± 24	0.003
3	315± 32	234 ± 18	0.019
8	290 ± 6	244 ± 13	0.005
24	321 ± 3	242 ± 6	< 0.001

Supplement 4: Insulin concentration in both groups

Table S2. Insulin concentration in both groups over two weeks.

Time (day)	Insulin/vildagliptin-eluting PLGA group, mU/mL	Insulin/PLGA group, mU/mL	*p* value
1	6.1 ± 1.2	5.7 ± 1.7	0.789
2	3.9 ± 0.8	3.1 ± 1.0	0.344
3	5.6 ± 0.3	4.8 ± 0.6	0.081
7	6.3 ± 1.2	8.5 ± 0.8	0.059
14	6.2 ± 1.0	6.8 ± 0.9	0.469

Supplement 5: Blood sugar in both groups

Table S3. Blood sugar in both groups in day 0, 7, and 14.

Time (day)	Insulin/vildagliptin-eluting PLGA group, mg/dl	Insulin/PLGA group, mg/dl	*p* value
0	419 ± 76	430 ± 36	0.731
7	426 ± 53	421 ± 29	0.854
14	430 ± 53	440 ± 114	0.827
